# *SHOX* gene and conserved noncoding element deletions/duplications in Colombian patients with idiopathic short stature

**DOI:** 10.1002/mgg3.39

**Published:** 2013-10-14

**Authors:** Gloria Tatiana Vinasco Sandoval, Giovanna Carola Jaimes, Mauricio Coll Barrios, Camila Cespedes, Harvy Mauricio Velasco

**Affiliations:** 1 Institute of Genetics, School of Medicine, Master in Human Genetics, Universidad Nacional de ColombiaColombia; 2 Department of Pediatrics, Hospital Militar CentralColombia; 3 Department of Pediatrics, Hospital De La Misericordia, Universidad Nacional De ColombiaColombia; 4 Department of Pediatrics, Hospital San Ignacio, Pontificia Universidad JaverianaColombia

**Keywords:** Idiopathic short stature, Latin America, mutations, regulatory regions, *SHOX*

## Abstract

*SHOX* gene mutations or haploinsufficiency cause a wide range of phenotypes such as Leri Weill dyschondrosteosis (LWD), Turner syndrome, and disproportionate short stature (DSS). However, this gene has also been found to be mutated in cases of idiopathic short stature (ISS) with a 3–15% frequency. In this study, the multiplex ligation-dependent probe amplification (MLPA) technique was employed to determine the frequency of *SHOX* gene mutations and their conserved noncoding elements (CNE) in Colombian patients with ISS. Patients were referred from different centers around the county. From a sample of 62 patients, 8.1% deletions and insertions in the intragenic regions and in the CNE were found. This result is similar to others published in other countries. Moreover, an isolated case of CNE 9 duplication and a new intron 6b deletion in another patient, associated with ISS, are described. This is one of the first studies of a Latin American population in which deletions/duplications of the *SHOX* gene and its CNE are examined in patients with ISS.

## Introduction

Short stature is defined as a height that is less than 2 standard deviations (SD) or one that is below the third percentile for the age and gender in a population (Wit et al. 2008). This includes both the pathological short height and short height considered a variant of normality. The short stature variants of normality are responsible for 80% of the hypogrowth and are caused by a lower genetic growth potential (familiar short stature), a delay in maturation (constitutional delay of growth and puberty), or to a combination of both processes (Aguirrezabalaga and Pérez 2006).

Two to three percent of the children in the world have short stature (Chen et al. 2009) and in 80% of the cases, there is no history of small for gestational age, growth hormone (GH) deficiency, or other pathologies. This group of patients has idiopathic short stature (ISS) (Wit et al. 2008).

Short stature homeobox (*SHOX* gene MIM 312865) is one of the main genetic contributors to human growth. Mutations cause Leri Weill dyschondrosteosis (LWD) and Langer mesomelic dysplasia. Short stature in Turner syndrome is the result of *SHOX* gene haploinsufficiency (Ellison et al. 1997; Flanagan et al. 2002). In 1997, Rao et al. were the first to describe *SHOX* gene mutations in individuals with ISS (ISS MIM 300582). After this study was published, several clinical and molecular studies identified the *SHOX* gene as the cause of the short stature phenotype (Huber et al. 2006). Intragenic mutations are described in 2–15% of cases (Jorge et al. 2008). Recently, it has been proven that the conserved noncoding elements (CNE) downstream of the *SHOX* gene regulate gene function (Sabherwal et al. 2007). These conserved elements present a 250 kb downstream regulatory domain that may be deleted in patients with ISS and in other *SHOX* gene defects with a frequency of 22% (Chen et al. 2009).

There is no evidence of the molecular behavior of the intragenic regions and of the CNE in Colombians. In Latin America, there is only one Brazilian report that describes molecular alterations in the *SHOX* gene. Therefore, the purpose of the present study is to examine alterations of the *SHOX* gene in patients with ISS in Colombia. The analysis was done using multiplex ligation-dependent probe amplification (MLPA).

## Material and Methods

Transversal descriptive study of 62 individuals with ISS was performed. They were enrolled between September 2010 and December 2012 and referred by the pediatric endocrinology outpatient services of five centers in Colombia (hospital sample frame) under the prior approval of the ethic committee of each institution. Their ages ranged from 2 to 16 years of age. Patients with GH deficiency, GH resistance, hypothyroidism, chronic diseases (cardiac, renal, pulmonary, genetic, gastrointestinal diseases, malnutrition, neoplasias, immunodeficiencies), constitutional delay of growth and puberty, small for gestational age, and intrauterine growth retardation were excluded. Bone dysplasia was excluded by X ray and clinical examination was done by a pediatric endocrinologist and geneticist. Skeletal anomalies such as bowing of the radius, dislocation of the ulna (anomalies of forearm), and malformation or absence of the fibula (lower leg deformities) were also eliminated for genetic analysis.

After an informed consent was signed, a blood sample was collected. DNA extraction was done using a MoBio Ultraclean Blood DNA Isolation Kit (Carlsbad, CA) and following the instructions of the manufacturer. The MLPA technique was done twice using an MRC Holland kit for *SHOX* and its CNE (SALSA MLPA KIT P018-E1 SHOX and P018-F1 updated version, Willem Schoutenstraat 6, Amsterdam, the Netherlands). This kit has probes for the pseudoautosomic region of the sex chromosomes. Some of these probes are directed to each exon of the *SHOX* gene. One was placed in the area before the promoter region and several others were placed to detect sequences downstream of *SHOX*. During the pretreatment and description phases, ABI 310 was used. MRC Holland Coffalyser v 9.4 Software was employed to analyze the results. This software calculates the odds ratios for each probe of every patient. An odds ratio of less than 0.7 was considered a deletion and an odds ratio of more than 1.3 was considered a duplication. All patients were compared with the DNA from three controls (subjects of normal size). Afterward, the Primer3 tool (http://frodo.wi.mit.edu/) was used to create flanking primers in order to confirm the deletions/duplications found. Additionally, parental DNA was analyzed by MLPA to identify the mutation origin (maternal or paternal inheritance or de novo). To confirm the new mutations, the Nimblegen platform, Roche Human CGH 385K Chromosome X Tiling Array, and Human CGH 385K Chromosome X Tiling Array (Madison, WI) were used. Statistical analysis of demographic and phenotypic variables was done using SPSS V20 (IBM, Madison Avenue, New York, NY).

## Results

Sixty-two patients between the ages of 2 and 16 were studied. The majority came from Bogotá, the capital of Colombia (78%). The ethnic origin of the Colombian individuals with ISS was broken down as follows: Caucasian 50%, mixed 43.6%, Amerindian 4.8%, and Black 1.6%. The gender and height distribution are described on Table 1. The proportionate short stature was defined by an endocrinologist using measurements such as armspan, top segment, and lower segment (data shown on Table 1).

**Table 1:** Anthropometric description of patients separated by gender

	Mean ± SD	
	Girls (*n* = 39)	Boys (*n* = 23)
Chronological age (years)	11.7 ± 5.6	10.4 ± 5.7
Mean standard deviation of height	−2.5 ± 1	−2.97 ± 1
Upper segment (cm)	63.7 ± 10.6	61.7 ± 11.7
Lower segment (cm)	60 ± 10.3	59.1 ± 11.9
Rate of proportionate short stature	1.1 ± 0.1	1 ± 0.1
Arm span (cm)	122.3 ± 20.8	120.1 ± 23
Age short stature diagnosed	5.17 ± 4.41	4.21 ± 6.2
Mid parental height	169.3 ± 20.8	164.6 ± 21.7

**Figure 1:**
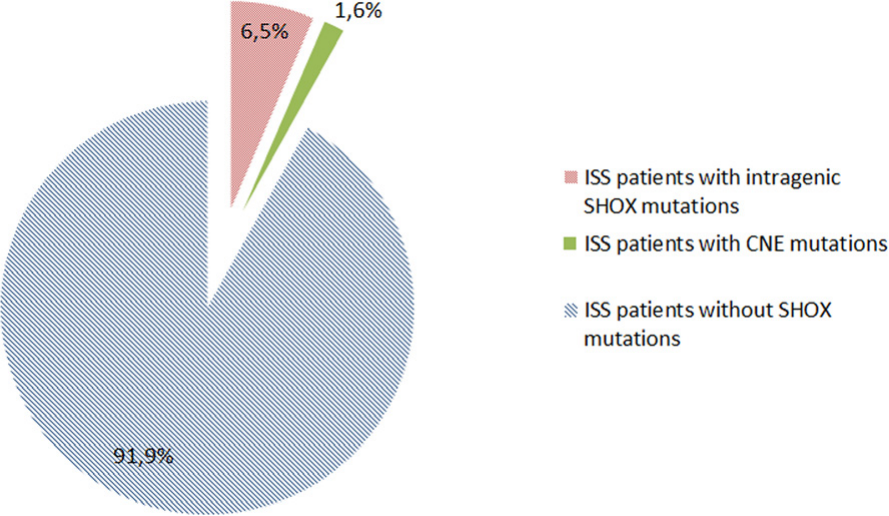
Proportion of mutations based on location on the gene found in patients with ISS.

Of 62 patients analyzed, two women and two men presented deletion from intragenic regions. One man had a duplication at the end of the regulatory region of the *SHOX* gene. This duplication included CNE 9 (Fig. 1). These results established an 8.1% frequency of alterations to the *SHOX* gene in Colombian patients with ISS (Table S1, Coffalyser report sheet). Phenotypical leftacteristics of five patients with mutations are described in Table 2.

**Table 2:** Phenotypical, molecular and inherited, and de novo mutations description of five patients with *SHOX* gene abnormalities

	Patients				
	ISS 02	ISS 06	ISS 14	ISS 38	ISS 39
Gender	M	M	F	M	F
Chronological age (years)	14	16	11	7	12
Gestational age (weeks)	40	40	40	40	40
Birth size (cm)	NR	NR	52	50	NR
Birth weight (g)	NR	NR	3000	2850	NR
Height (cm)	144	151	127.8	104.5	135.5
Height SDS	−3.05	−2.82	−2.6	−3.44	−2.82
Weight (kg)	32	52	30.4	16	36.1
Mid parental height	162.5	162	154.5	144.5	168.5
Disproportionate short stature	Yes	Yes	Yes	Yes	Yes
Age at diagnosis of ISS (years)	12	2	5	4	11
MLPA result	Del PAR1 probe 8	Del PAR1 probes 2–3	Del PAR1 probe 4	Del PAR1 probe 10	Gain PAR1 probes 22–23
Genic position	Exon 6	UTR 3′ Exon 1	Exon 2	Intron 6b	CNE 9
Inherited mutation	No/no data from father^1^	Yes/paternal route	Yes/maternal transmission	De novo	Yes/paternal transmission
Father height (cm)	165^2^	161	168	162	152^2^
Mother height (cm)	147	150	154	162	150

1 Deceased father.

2 Referred by the mother.

Patients with short stature associated with *SHOX* gene abnormalities may have different clinical expressions. These manifestations range from proportionate short stature to LWD 7. In our study, patients with *SHOX* gene alterations did not present statistically significant differences with respect to parameters such as age and the standard deviation of height compared to ISS patients who did not present *SHOX* gene alterations.

**Figure 2:**
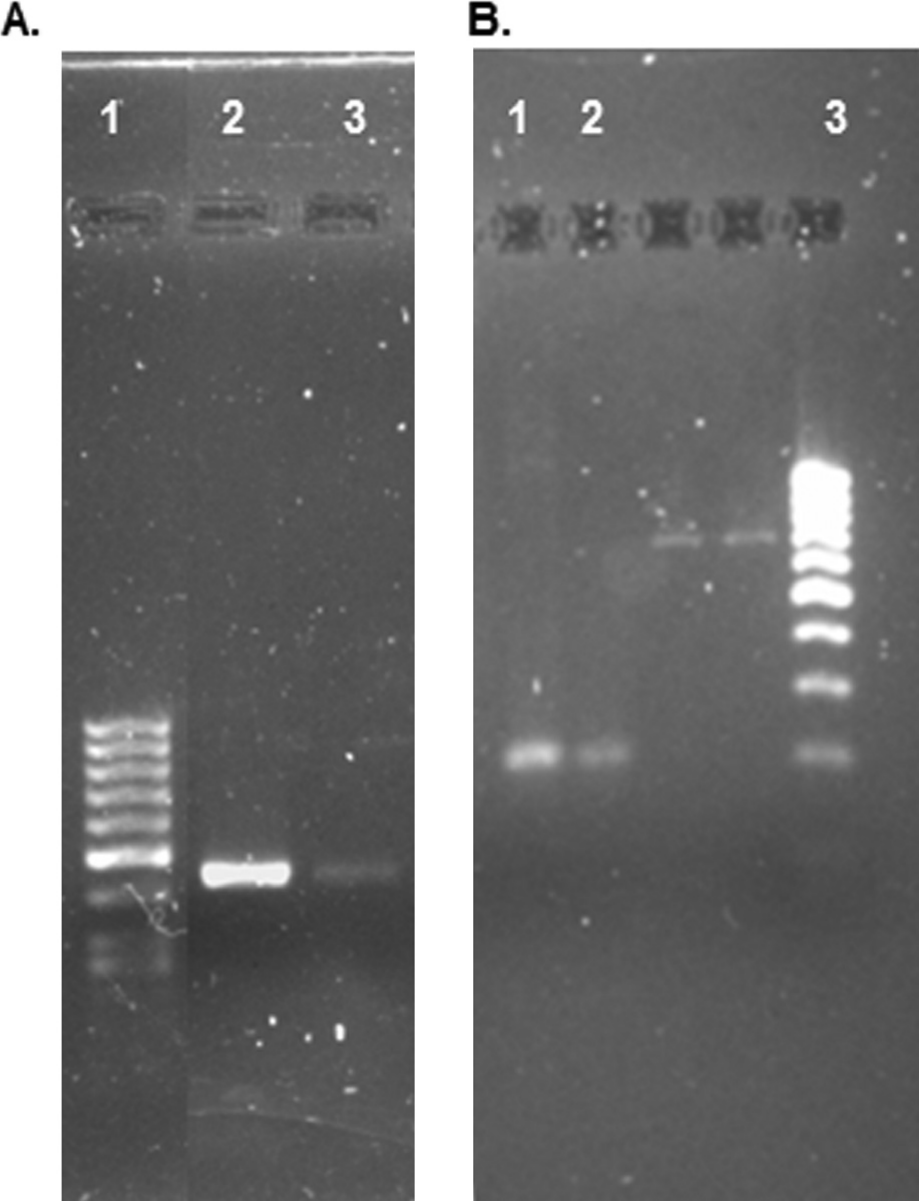
Amplified exons 6 (patient ISS2) and 2 (patient ISS 38) in agarose gel electrophoresis. Agarose gel for patient ISS2, (A) lane 1: ladder 100 bp, lane 2: normal reference, lane 3: patient ISS2. (B) Lane 1: normal reference, lane 2: ISS 38 patient, lane 3: ladder.

Of patients with deletions in the intragenic regions, patient ISS 02 had a loss of heterozygosity (LOH) in the exon 6 region. Patient ISS 38 had LOH in exon 2. Both results were confirmed by qualitative reduction of exons 6 and 2 in electrophoresis gel (Fig. 2).

Patient ISS 14 had deletion of exon 1 including part of the 5′ UTR region of the gene while LOH of 6b intron was reported for patient ISS 39. Finally, patient ISS 06 had an area gain which corresponds to CNE 9. This area is part of the regulatory region of *SHOX* gene (Table 2). No qualitative pattern of signal reduction was observed by amplification of adjacent regions in patients ISS14 and ISS 39. No data are available.

**Figure 3:**
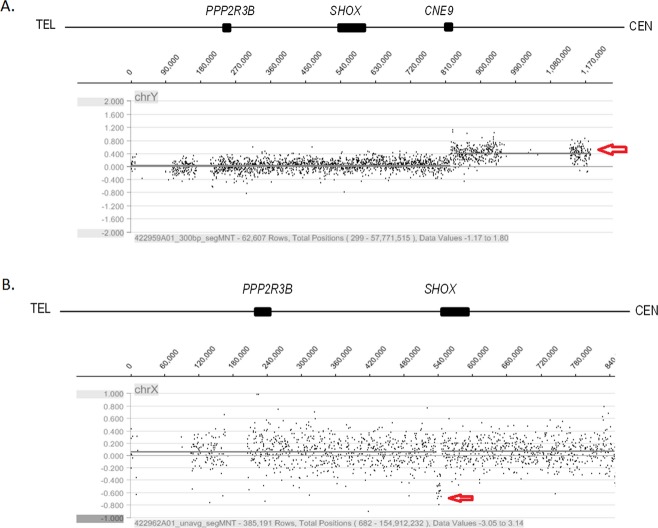
Fine-tiling X and Y chromosomes aCGH mutation description (Nimblegen Y chromosome-specific array). Fluorescence intensity log2 ratios were calculated based on reference values and are shown for each oligonucleotide from telomere to centromere on the short arm of chromosomes X and Y. Sequence coordinates are taken from the X and Y chromosomes assembly (NCBI Assembly GRCh 37p). Log2 ratio data are shown in NimbleGen SignalMap data viewer software. Each dot indicates the midpoint of 300 bp and is calculated by 1–15 probes. A log2 ratio value above 0.5 was considered duplication. A log2 ratio value of less than −0.5 was considered a deletion. Red arrows show the mutation location and correspond to: 362 kb duplication from ∼822,043 to ∼1,184,009 bp on the short arm of the Y chromosome (NCBI assembly GRCh37p) in the CN9 region (A) and 8.2 kb deletion from ∼536,508 to ∼544,722 bp on the short arm of the X chromosome (NCBI assembly GRCh37p). This region corresponds to exon 1 (ISS 14) (B).

CGH 385k Chromosome X Tiling array and Human CGH 385K Chromosome Y Tiling array with Median Probe Spacing of 340 and 20 bp, respectively, were used to confirm the results of new mutations (ISS 6, CNE 9; ISS 14, exón 1; ISS 39, intron 6b) obtained from MPLA. After these confirmations, there are two new *SHOX* gene mutations that have not been reported before: a duplication of close to 362 kb in the CN9 region (from ∼822,043 to ∼1,184,009 bp) in patient ISS 06 and a deletion of approximately 8.2 kb in exon 1 (from ∼536,508 to ∼544,722 bp) in patient ISS 14 (Fig. 3).

In the case of all five patients with mutations, the origin and type of segregation were analyzed by doing an MLPA of the parents. Patients ISS 06 and ISS 39 presented paternal inheritance. Patient ISS 14 had maternal transmission and patient ISS 38 mutation was de novo (Table 2).

## Discussion

Longitudinal growth is a complex, continuous, nonlinear process, and is determined by genetic factors and modulated by both permissive and regulator factors (Iughetti et al. 2010).

Our study of 62 patients with ISS showed some mutations in five patients that explained their short stature. When the group of patients with *SHOX* gene mutations and the group with no mutations are compared, no statistically significant differences were found regarding the standard deviation of height, mid parental height, arm span, upper segment, lower segment, and age of the short stature diagnosis. This confirms the fact that in patients with ISS, the presence of a phenotype–genotype correlation is difficult to find (Rappold et al. 2007). Several studies showed decreased cortical Volumetric Bone Mineral Density and cortical thickness and enlarged diaphysis in patients with isolated *SHOX* deficiency, which suggested that *SHOX* haploinsufficiency could cause leftacteristic skeletal anomalies at the radius (Soucek et al. 2013). This leftacteristic was not evaluated in our study.

Within the last few years, the haploinsufficiency of the SHOX gene has been described as one of the most important causes of short stature and that, for two out of three patients with haploinsufficiency, the cause is deletions that are intragenic and regulator region in type (Funari et al. 2010).

*SHOX* gene locus and adjacent regions are prone to deletions/duplications because of the high incidence of repeated sequences throughout PAR1 (May et al. 2002). This situation predisposes to a nonallelic homologous recombination resulting in a high percentage of recombinant fractions (Fukami et al. 2008). These fractions may be assessed by Fluorescence in situ hybridization, MLPA, or array Comparative Genomic Hybridization (aCGH). In this study, MLPA and aCGH techniques were used to establish the frequency of changes in the number of copies of *SHOX* gene in Colombian patients with ISS. This frequency was 8.1%, which fit the 2–15% data reported in the literature (Binder 2010). The study done by Rappold et al. 2002; of a large cohort of 1534 patients with ISS reported a mutational frequency of 2.2% (nine point mutations and 25 deletions) (Rappold et al. 2002) while Huber et al. 2006; found a frequency of 15% in 84 patients (Huber et al. 2006). It is possible that the frequency found in our population may be higher when point mutations are analyzed.

In 2008, Jorge et al. found a 3.2% frequency of point mutations in 63 patients. This is the first Latin American study of *SHOX* gene mutations in ISS. No deletions or duplications were studied. In another study, Rodríguez et al. (2013), using MLPA and sequencing techniques, confirmed mutations in *SHOX* gene in seven children with LDW syndrome. ISS were excluded. In our study, the frequency of deletion/duplication type mutations analyzed by MLPA was 2.5 times higher with a relatively similar sample size which suggests that our patients have a different mutational frequency. Gene sequencing is needed to analyze the behavior of point mutations in our population.

With respect to mutations found in the present study and those previously reported in literature, the deletion of a fragment of 204 bp in exon 2 of the *SHOX* gene was detected. This deletion was found with probe 01146-L06220 of MLPA. Grigelioniene et al. (2001) reported a deletion of 11 bp in this same region in a patient with LWD. The deletion this group reported was a frameshift type related to a codon stop in position 74 of the amino acid sequence. This exon has interaction functions with SOX 9 during chondrogenesis because the exon encodes for a region that the N-terminal domain of the SHOX protein consists of and contains alternative promoter elements that define the amount of functional protein generated from the *SHOX* gene (Aza-Carmona et al. 2011). Likewise, Aza-Carmona et al. showed that, using a mutated *SHOX* construct in regions other than homeodomain, the interaction with SOX9 diminished and caused short stature.

Another recurrent mutation was a deletion of a 231-bp fragment (MLPA 09337-L00911 probe) in exon 6 (patient ISS 02) corresponding to a portion of the region that encodes the C-terminal domain of the SHOX protein. According to Aza-Carmona et al., this domain also participates in the SOX9 interaction (Aza-Carmona et al. 2011). Deletions of this exon have been reported in patients with ISS by Rappold et al. previously. This group described a loss of four amino acids in a region very close to the deleted MLPA probe (Rappold et al. 2002).

Furthermore, three new mutations which had not been previously reported are described. The first one is a deletion in exon 1 that was transmitted by the maternal X chromosome. It was verified by a specific MLPA probe and was confirmed with aCGH. A loss of a portion of *SHOX* gene 5′UTR region (8.2 kb) was seen here. This sequence can form highly stable secondary structures and contains a terminal oligopyrimidinic tract and seven AUG codons upstream of the open reading frame. These regions are 44–67% similar to Kozak's consensus sequences. All these are key elements at the translation level (Blaschke et al. 2003).

Another new mutation found was the loss of at least 392 bp (MLPA 09338-L15503 probe) in 5′UTR of intron 6b in patient ISS39, which was inherited from the father (paternal height 152 cm). Despite having found the same mutation in the same region four times in this family, these findings could not be duplicated by high density array because its probes are located 700 bp from the above mentioned microdeletion and surround it. Although this deletion is in a region that hypothetically is not involved in gene expression nor in alternative splicing and is not reported in the LOVD database (http://grenada.lumc.nl/LOVD2/MR/variants_statistics.php) (Fokkema et al. 2005), both subjects (father and daughter) have ISS.

Finally, an atypical case of a major duplication of 362 kb in the CNE 9 region is reported (∼822,043 to ∼1,184,009 bp) in a male patient with proportionate short stature (151 cm, −2.82 SD) without X trisomy nor intragenic *SHOX* mutations. Father and son share this genic trace and have short stature.

As was mentioned by Thomas et al. (2009), there is a high phenotypical variability related to gain or loss of *SHOX* gene sequences and their regulatory regions. When duplications are analyzed, these duplications have been described in trisomies or in large structural anomalies (duplication type) that are generally associated with tall stature. However, there are also several reports of isolated *SHOX* gene duplications and normal stature, but these cases also include Madelung deformity (Grigelioniene et al. 2001), complex rearrangements with deletion and duplication of *SHOX* in the same patient with short stature (second case reported by Thomas et al.), or proportionate short stature with isolated microtrisomy of *SHOX* (Iughetti et al. 2010). None of these reported cases is specifically affected by CNE 9.

Fukami et al. (2006) demonstrated that CNE 9 (ECS4) is a potential binding site for HOXA9, HOXB9, PBX1, MEIS1 proteins, and for complexes like PBX1-HOXA9 and MEIS1-HOXA9. These complexes are necessary to skeletal development. Based on this, we suggest that duplication of CNE 9 could produce an imbalance in the regulation of mechanisms that promote transcription of the *SHOX* gene. This hypothesis must be tested in in vitro studies.

In summary, different mutations were detected in a group of Colombian patients with ISS. These mutations compromised both intragenic and CNE regions and some of these must be studied in vitro to determine their impact on the development of this phenotype.

## Supporting Information

Table S1 Prediction Coffalyser report sheet to ISS patients with *SHOX* mutations in this study.
