# Supplementary material for: SHOX gene and conserved noncoding element deletions/duplications in Colombian patients with idiopathic short stature
Source: Mol Genet Genomic Med. 2013 Oct 14;2(2):95–102. doi: 10.1002/mgg3.39 (PMC3960050; doi:10.1002/mgg3.39)
Supplement: Table S1 — Prediction Coffalyser report sheet to ISS patients with SHOX mutations in this study. [file mgg30002-0095-sd1.docx]

Supplementary Table 1: prediction Coffalyser report sheet to ISS patients with *SHOX* mutations in this study.

| **Position** | **Length** | **Gene** | **OR** | **OR** | **OR** | **OR** | **OR** |
| --- | --- | --- | --- | --- | --- | --- | --- |
|  |  |  | ISS 02 | ISS 06 | ISS 14 | ISS 38 | ISS 39 |
| Xp22.33 | 211 | X-000.2 PPP2R3B | 0,81 | 0,81 | 1,2 | 1,11 | 1,01 |
| Xp22.33 | 266 | X-000.5 LOC159015 | 1,14 | 1,14 | 0,68 | 1,17 | 1,1 |
| Xp22.33 | 166 | X-000.5 SHOX Exon 01 | 0,88 | 1,05 | 0,68 | 0,95 | 0,96 |
| Xp22.33 | 204 | X-000.5 SHOX Exon 02 | 1,21 | 0,94 | 0,87 | 0,52 | 1,07 |
| Xp22.33 | 245 | X-000.5 SHOX Exon 03 | 1,15 | 1,15 | 0,8 | 1,04 | 1,03 |
| Xp22.33 | 300 | X-000.5 SHOX Exon 04 | 1,13 | 1,13 | 0,82 | 0,98 | 0,97 |
| Xp22.33 | 337 | X-000.5 SHOX Exon 05 | 0,73 | 0,88 | 1,3 | 1,13 | 1,09 |
| Xp22.33 | 231 | X-000.5 SHOX Exon 06 | 0,56 | 0,83 | 1,24 | 1,06 | 0,97 |
| Xp22.33 | 226 | X-000.5 SHOX Intron 06A | 1 | 0,87 | 1,12 | 0,96 | 0,97 |
| Xp22.33 | 392 | X-000.5 SHOX Intron 06B | 0,82 | 0,94 | 1,24 | 0,98 | 0,53 |
| Xp22.33 | 136 | X-000.5 SHOX-area | 1,01 | 0,95 | 1,04 | 1,07 | 0,98 |
| Xp22.33 | 154 | X-000.5 SHOX-area | 1,07 | 1,11 | 0,79 | 1,01 | 1,04 |
| Xp22.33 | 172 | X-000.5 SHOX-area | 1 | 0,95 | 1 | 0,99 | 0,98 |
| Xp22.33 | 199 | X-000.5 SHOX-area | 0,82 | 1,12 | 1,13 | 1,02 | 1 |
| Xp22.33 | 318 | X-000.5 SHOX-area | 0,92 | 1 | 1,16 | 0,97 | 0,93 |
| Xp22.33 | 432 | X-000.5 SHOX-area | 0,93 | 1,1 | 1,08 | 0,97 | 1,1 |
| Xp22.33 | 463 | X-000.5 SHOX-area | 1,09 | 0,86 | 1,25 | 1,03 | 1,08 |
| Xp22.33 | 290 | X-000.5 SHOX-area | 0,9 | 1,04 | 0,85 | 1 | 0,95 |
| Xp22.33 | 185 | X-000.5 SHOX-area | 0,96 | 0,93 | 0,9 | 0,98 | 0,96 |
| Xp22.33 | 148 | X-000.5 SHOX-area | 1 | 1,04 | 0,9 | 1,07 | 1,04 |
| Xp22.33 | 178 | X-000.5 SHOX-area | 0,99 | 1,03 | 0,8 | 0,88 | 0,94 |
| Xp22.33 | 442 | X-000.5 SHOX-area | 0,85 | 1,36 | 0,8 | 0,89 | 1,09 |
| Xp22.33 | 377 | X-000.5 SHOX-area | 0,92 | 1,58 | 0,93 | 0,99 | 1,07 |
| Xp22.33 | 403 | X-001.3 CRLF2 | 0,86 | 1,12 | 0,9 | 1 | 0,94 |
| Xp22.33 | 386 | X-001.4 CSF2RA | 1,14 | 0,91 | 0,87 | 1,07 | 0,96 |
| Xp22.33 | 142 | X-001.4 IL3RA | 0,99 | 0,96 | 0,89 | 1 | 1,01 |
| Xp22.33 | 310 | X-001.7 ASMT | 0,74 | 0,87 | 1,29 | 1,07 | 1 |
| Xp22.33 | 453 | X-002.4 ZBED1 | 0,86 | 1,08 | 1,25 | 1,03 | 1,07 |
| Xp22.33 | 254 | X-003.0 ARSF | 0,88 | 0,97 | 0,87 | 1,03 | 1,08 |
| Xp22.33 | 328 | X-003.6 PRKX | 1,05 | 0,97 | 1,06 | 0,92 | 1,02 |
| Xp22.31 | 283 | X-006.0 NLGN4X | 0,78 | 1,17 | 0,7 | 1,04 | 0,98 |
| Xp22.31 | 238 | X-008.6 KAL1 | 1,29 | 0,93 | 1,07 | 1,01 | 0,96 |
| Xp22.2 | 274 | X-014.8 FANCB | 0,92 | 1,05 | 0,75 | 0,95 | 0,96 |
| Xq25 | 420 | X-129.1 AIFM1 | 0,92 | 0,9 | 1,09 | 0,96 | 1,08 |
| Xq28 | 355 | X-154.8 VAMP7 | 0,88 | 1,01 | 0,77 | 0,96 | 0,92 |
| 01p22 | 160 | C | 1,12 | 1 | 0,71 | 0,96 | 0,97 |
| 01q32 | 346 | C | 0,92 | 0,94 | 0,9 | 1,05 | 1,01 |
| 04p16 | 191 | C | 0,75 | 0,98 | 0,9 | 0,93 | 1,01 |
| 05q31 | 130 | C | 1,07 | 1,14 | 1,11 | 1,15 | 1,01 |
| 09q31 | 490 | C | 1,21 | 0,86 | 1,23 | 0,95 | 1,05 |
| 13q14 | 219 | C | 1,05 | 1,1 | 0,73 | 0,95 | 0,88 |
| 15q21 | 412 | C | 0,92 | 0,83 | 0,75 | 1,01 | 1,01 |
| 16p13 | 369 | C | 1,1 | 1,19 | 0,84 | 1,04 | 1,08 |
| 16p13 | 474 | C | 0,9 | 0,98 |  | 1,01 | 1 |
| 18q21 | 261 | C | 1,22 | 1,05 |  | 0,98 | 1 |

OR: ODDS RATIO

|  | Normal |  | LOH < 0,7 |  | Duplication > 1,3 |
| --- | --- | --- | --- | --- | --- |
